# Tocilizumab Contributes to the Inflammatory Status of Mature Dendritic Cells through Interleukin-6 Receptor Subunits Modulation

**DOI:** 10.3389/fimmu.2017.00926

**Published:** 2017-08-16

**Authors:** Daniel Meley, Audrey Héraud, Valerie Gouilleux-Gruart, Fabrice Ivanes, Florence Velge-Roussel

**Affiliations:** ^1^EA 4245 Cellules Dendritiques, Immuno-modulation et Greffes, Université François-Rabelais de Tours, UFR de Médecine, Tours, France; ^2^CNRS UMR 7292; Université François-Rabelais de Tours, UFR de Médecine, Tours, France; ^3^Department of Immunology, CHRU de Tours, Tours, France; ^4^Service de Cardiologie, CHRU de Tours, Tours, France; ^5^UFR des Sciences Pharmaceutiques, Tours, France

**Keywords:** dendritic cell, tocilizumab, IL-6 receptor subunits, rheumatoid arthritis, inflammation

## Abstract

Tocilizumab, a humanized anti-IL-6 receptor α (IL-6Rα) is widely used in the treatment of a panel of pathologies such as adult and juvenile rheumatoid arthritis (RA) and the systemic form of juvenile idiopathic arthritis in children. Its indications are expected to be largely extended to other inflammatory diseases in close future. Dendritic cells (DCs) appear to be deeply involved in the immunopathology of these diseases, yet the effects of tocilizumab on these cells were poorly studied. In this study, we explored the effect of tocilizumab on the regulation of IL-6R subunits [gp130, soluble form of IL-6Rα (sIL-6Rα), and mIL-6Rα] in human monocyte-derived DCs. Human DCs were derived from CD14^+^ monocytes purified with beads with IL-4 and granulocyte macrophage colony-stimulating factor. *Ex vivo* cultures of DCs were performed in the presence of tocilizumab. Using lipopolysaccharide (LPS) maturation of DCs, we demonstrated that tocilizumab did not inhibit IL-6 secretion, enhanced mIL-6Rα expression, and largely increased sIL-6Rα secretion. MAPK modulated STAT3 phosphorylation and surface expression of IL-6Rα in LPS-DCs. Tocilizumab had no impact on STAT3 phosphorylation in LPS-DCs while both LPS and IL-6 increased its activation. Tocilizumab modulated the regulation of IL-6R subunits leading to an inflammatory status of DCs and a massive secretion of IL-6Rα. Our results demonstrate that DCs acquire a pro-inflammatory profile following tocilizumab treatment, becoming a major source of IL-6 *trans*-signaling activation that might explain the poor clinical benefit in some RA patients.

## Introduction

Dendritic cells (DCs) are highly specialized antigen presenting cells (APC) that are able to induce both specific immunity and immune tolerance (1). Using the information gathered from the tissue where they reside, DCs adjust their functional activity to ensure that protective immunity is favored while unwanted or exaggerated immune responses are prevented. Upon stimulation, DCs undergo various phenotypic changes among which an increase in CMHI/CMHII-dependent antigen presentation and expression of co-stimulatory molecules or chemokine receptors that induce migration of the primed DCs toward the lymphoid nodes where they activate the adaptive immune response (2).

According to their origin and homing (non-lymphoid or lymphoid tissue), DCs are a heterogeneous group of cells, with each subtype having specific functions and properties, as shown in murine models as well as in human (3–5).

The ability of monocytes to differentiate in DCs was first described by Sallusto and Lanzavecchia who reported the generation of DCs from human peripheral blood monocytes after a 7-days *in vitro* culture with granulocyte macrophage colony-stimulating factor (GM-CSF) and interleukin (IL)-4 (6). The close link between *in vitro* generated monocyte-derived DCs (moDCs) and inflammatory DCs found *in vivo* was confirmed by transcriptome analyses (7). Consequently, this model of moDCs has been used during the last 20 years, contributing to the understanding of DCs and the development of their use in cell therapy.

While little is known regarding the roles of the different DC subsets in inflammatory diseases, e.g., in rheumatoid arthritis (RA), inflammatory DCs were found in blood as well as in lymph nodes in RA patients (7). Moreover, both myeloid and plasmacytoid DCs populations are increased in synovial fluid in RA patients compared with peripheral blood (8). Myeloid CD1c^+^ DCs appear to play a major role in promoting synovial inflammation through the production of large amounts of T cell-attracting chemokines and the pro-inflammatory cytokines IL-12 and IL-23, which promote T cell differentiation in IFN-γ-producing type 1 (Th1) and IL-17-producing type 17 (Th17) cells, respectively (9). In RA patients, the promotion of Th17 phenotype was also linked to increased levels of IL-6, a key factor in the physiopathology of inflammatory diseases in general, and RA in particular (10). IL-6 is the founder of a family of eight cytokines (including IL-11, LIF, or CT-1) (11), which receptors share the common subunit gp130. This membrane subunit bears the capacity to activate the JAK/STAT3 pathway, inducing the biological response to IL-6. The IL-6 system bears two distinct signaling mechanisms: the *cis* and the *trans*-signaling. The conventional *cis* signaling involves both membrane bound gp130 and IL-6 receptor α (mIL-6Rα). The *trans*-signaling still requires a membrane gp130 that interacts with a soluble form of IL-6Rα (sIL-6Rα), which is produced either through alternative splicing or membrane shedding by the ADAM17 protease (12). The mIL-6Rα is present at the surface of both immune (including monocytes and DCs) and non-immune cells (e.g., hepatocytes) (13), whereas gp130 is ubiquitously expressed in all cell types. Thus, the cells lacking mIL-6Rα but expressing gp130 can still respond to IL-6 stimulation through the *trans*-signaling, consequently enlarging the spectrum of IL-6 target cells. The regulation of IL-6R expression is poorly described and appears largely model dependent. Moreover, the receptor modulation in human DCs is mostly unknown. A thorough understanding of IL-6R regulation is of utmost importance as IL-6 is largely involved in immune regulation, not only in inflammation but also in the immune tolerance response. Interaction of IL-6 with the complex gp130 (dimer)/IL-6Rα triggers a signaling cascade involving Janus kinases that leads to the phosphorylation of STAT3. The STAT3 signaling pathway is the major intrinsic pathway modulating the inflammatory response in many cells, such as cancer and immune cells (14, 15). In DCs, when STAT3 is activated, the cells display tolerogeneic competences, retaining an immature phenotype and inhibiting the T cell response (16, 17).

The classic therapeutic strategy in RA patients consists in associating disease-modifying antirheumatic drugs, e.g., methotrexate, with the anti-TNF-α therapy. However, 25–30% of patients do not respond to the treatment or develop resistances and, thus, necessitate an alternative strategy (18, 19). As increased levels of circulating IL-6 are described, an alternative therapy is the use of the anti-IL-6Rα antibody tocilizumab (20). This therapeutic antibody is a humanized monoclonal antibody targeting both sIL-6Rα and mIL-6Rα (21). Clinical studies did not report significant adverse effects, apart from a few cases of mild hepatic disorders. Yet, it is now indicated as an alternative treatment to several other inflammatory and autoimmune diseases, such as lupus, Crohn’s disease, and Uveitis, in many countries (11). Betts et al. confirmed the capacity of tocilizumab to inhibit the IL-6-driven STAT3 pathway and showed that it had no impact on moDCs phenotype, but they did not evaluate its effect neither on inflammatory DCs that correspond to the disease conditions (22) nor on IL-6R expression.

We describe here for the first time the regulation of both IL-6R subunits during DC maturation as well as the impact of tocilizumab treatment on their expression.

## Materials and Methods

### Materials

Tween-20 was from Biosolve Chimie (Dieuze, France). All other chemicals were obtained from Sigma Aldrich (St. Louis, MO, USA). RPMI 1640, FCS, PBS, and Ficoll gradient were from Dominique Dutscher (Brumath, France). Lipopolysaccharide (LPS) was from Invivogen (Toulouse, France). IL-6, CD14-conjugated magnetic microbeads, rhGM-CSF, and rhIL-4 were from Miltenyi Biotec (Bergisch Gladbach, Germany). SB203580 was from Jena Biosciences (Jena, Germany). PD 98059 was from Enzo Life Sciences (Farmingdale, NY, USA). PE-conjugated mouse monoclonal anti-human IL-6Rα (clone 17506), APC-conjugated mouse monoclonal anti-human gp130 (clone 28126), and PE-conjugated and APC-conjugated mouse IgG1 isotype control (clone 11711) were from R&D Systems (Abingdon, UK). FITC-conjugated mouse anti-human CD83 (clone HB15e), APC-conjugated mouse anti-human CD25 (clone M-A251), and FITC-conjugated and APC-conjugated mouse IgG1 isotype control (clone MOPC-21) were from BD Biosciences (Franklin Lakes, NJ, USA). PE-conjugated mouse anti-human CD14 (clone RMO52), CD209/DC-SIGN (cloneA2ND1), CD80 (clone MAB104), and PE-conjugated mouse IgG1 and IgG2a isotype control (clone 679.1Mc7 and 7T4-1F5, respectively) were from Beckman Coulter. Phosphospecific antibodies against STAT3 (Tyr705) (clone 3E2), p38 MAPK (Thr180/Tyr182), p42/44 MAPK (Erk1/2) (Thr202/Tyr204), and antibodies against the total form of STAT3 (clone 79D7), p38 MAPK, p42/44 MAPK, and β-actin were from Cell Signaling Technologies (Danvers, MA, USA). Goat anti-rabbit and anti-mouse horseradish peroxidase antibodies were from Bio-Rad (Hercules, CA, USA). Protran BA83 nitrocellulose membrane and ECL Prime Western blotting detection reagent were from GEHealthcare (Little Chalfont, UK). Protease inhibitor cocktail was from Calbiochem (Darmstadt, Germany). Prestained protein marker VI was from Applichem (Darmstadt, Germany). BCA protein assay reagent and the F(ab′)2 preparation kit were from Pierce—Thermo Scientific (Waltham, MA, USA). Tocilizumab and rituximab were kindly provided by Pr. H. Watier (University of Tours).

### Generation of DCs from Peripheral Blood Mononuclear Cells (PBMCs)

Peripheral blood mononuclear cells were obtained from healthy volunteers according to institutional research protection guidelines (Agreement No CA-REC-2015-123). PBMCs were isolated by Ficoll™ gradient centrifugation (density 1.077). Monocytes were purified from PBMC by positive selection using CD14-conjugated magnetic microbeads and were differentiated in culture medium (containing 66 ng/ml rhGM-CSF and 25 ng/ml rhIL-4). On day 6, immature DCs were harvested, washed, and suspended in culture medium with IL-4 and GM-CSF or used for analysis in flow cytometry or Western blot. The immature DCs were treated with 50 ng/ml LPS (from *E. coli*) or 150 ng/ml IL-6 for the indicated time. Cells were harvested, washed, and used for analyses in flow cytometry or Western blot. When necessary, cells were pretreated for 30 min with 25 µM SB203580, 25 µM PD98059, TIMP-3, and 50 µg/ml tocilizumab or rituximab.

### Flow Cytometry Analysis

Dendritic cells were incubated at 4°C with saturating concentrations of fluorochrome-conjugated monoclonal antibodies (mAbs) (anti-CD14, anti-CD209/DC-SIGN, anti-C80, anti-CD83, anti-CD25, anti-IL-6Ra, and anti-gp 130), in the dark for 30 min, washed twice with PBS–SVF 5% and analyzed with a FACS Canto cell analyzer (Becton Dickinson, USA). Data were analyzed with Diva 6 Software. Data are represented as % of isotype MFI (specific MFI/isotype MFI × 100). When specified in legend; results are represented as % of control condition: (condition 1 specific MFI/condition 1 isotype MFI × 100)/(control condition specific MFI/control condition isotype MFI × 100).

### Immunoblotting

Protein extract and immunoblotting were performed as previously described using the mouse anti-phospho-STAT3, anti-phospho-p38 MAPK, anti-phospho-p42/44 MAPK, anti-STAT3, anti-p38 MAPK, anti-p42/44 MAPK, and anti-b-actin. Western blotting detection reagent was used for ECL reaction and the signal was detected and quantified using PiXi gel doc system (Syngene, Cambridge, UK). Regarding the Figure 6C,D, the same samples were loaded in parallel on two distinct gels and run in the same time in the same conditions. This was also the case for the transfer procedure. Membranes were initially probed overnight at 4°C with anti-p38-P (gel 1: 38 kDa) and anti-Erk1/2-P (gel 2: 42–44 kDa), then revealed with anti-primary antibodies linked to HRP. The size of Erk1/2 and p38 did not allow simultaneous analysis of the actin expression (42 kDa). After imaging, gels were harsh stripped, blocked as previously described and probed with anti-actin, rabbit anti-STAT3, and mouse anti-STAT3-P antibodies. Anti-actin was revealed with an anti-rabbit HRP antibody, while anti-STAT3 and STAT3-P were revealed with anti-rabbit-DyLight 800 nm and anti-mouse-HRP antibodies, respectively. Both actin bands were similar (gel 1 and 2) and we chose to illustrate here only the actin band of gel 2 for presentation reason. However, quantification was done relative to the correct respective actin band.

### Statistical Analysis

Histograms represent the mean ± SD. Statistical significance was determined by the unpaired non-parametric Mann–Whitney test. Difference was considered significant when *p* < 0.05. All the experiments were performed at least in triplicates.

## Results

### Both IL-6 and LPS Modulate IL-6R Subunit Expressions on DCs

**Figure 1:**
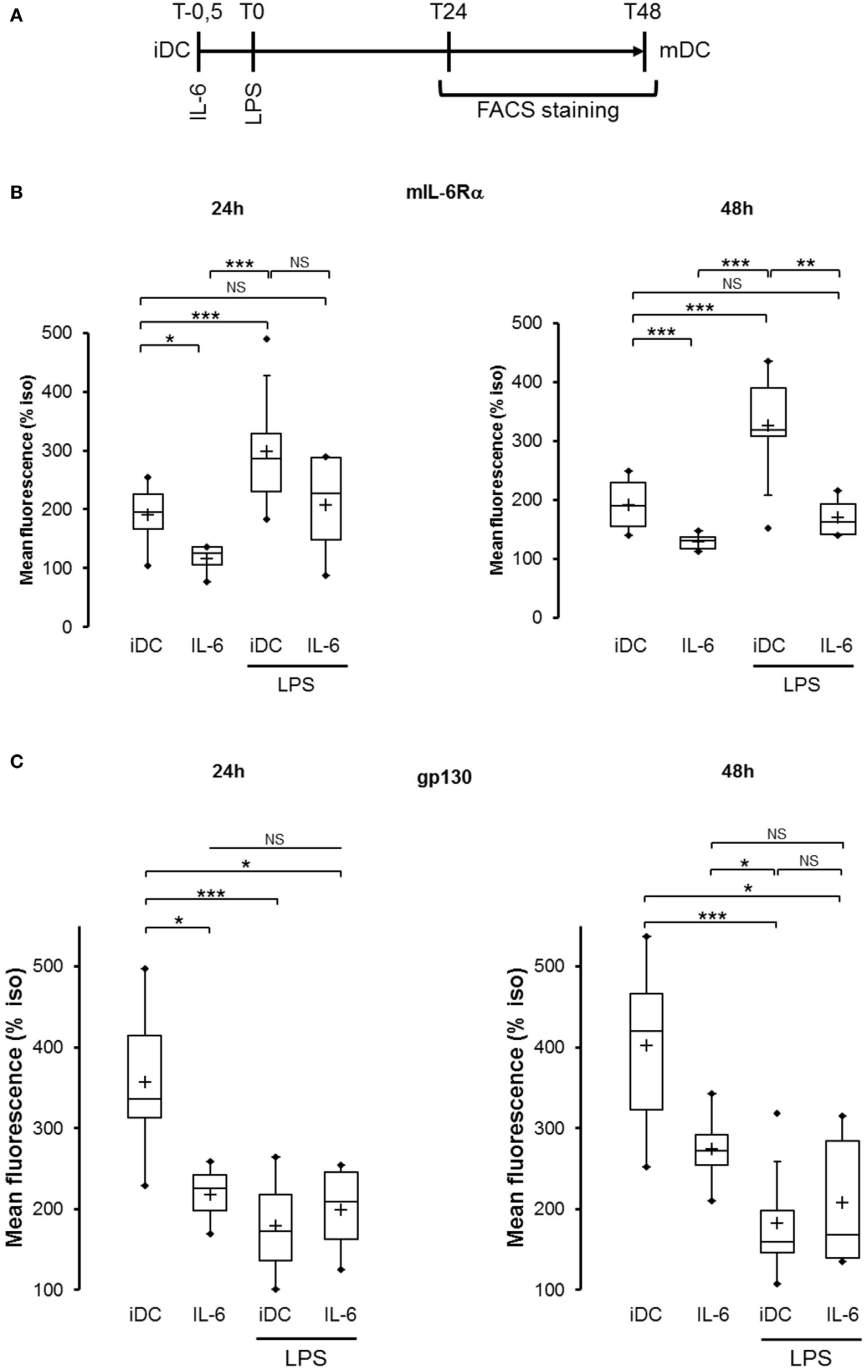
IL-6 inhibited both IL-6 receptor (IL-6R) subunits expression in immature and matured dendritic cells (DCs). **(A)** Flowchart: iDCs were pretreated for 30 min with IL-6 150 ng/ml prior to lipopolysaccharide (LPS) maturation (50 ng/ml). Matured DCs were harvested at 24 and 48 h and analyzed by flow cytometry. **(B)** iDCs were treated as described in **(A)** and mIL-6Rα expression was assessed (*n* ≥ 4). **(C)** iDCs were treated as described in **(A)** and membrane gp130 expression was assessed (*n* ≥ 4). “*,” “**,” and “***” indicate statistical differences with, respectively, *p* < 0.05, *p* < 0.01, and *p* < 0.001.

To clearly define the numerous parameters of the inflammatory DCs model, we first analyzed the expression of IL-6R subunits in the LPS maturation model of moDCs and the relationship between IL-6, LPS, and the modulation of IL-6R subunits (Figure 1A; Figure S1 in Supplementary Material). As expected, exogenous IL-6 induced a significant reduction of mIL-6Rα expression compared with LPS (*p* < 0.001) both at 24 and 48 h. Interestingly, cells co-treated with LPS and IL-6 displayed a lower mIL-6Rα level compared with the LPS alone condition (Figure 1B). Regarding gp130 regulation, IL-6 treatment inhibited gp130 expression to the membrane to a lower extent than LPS, and co-treatment with LPS and IL-6 did not display any cumulative inhibitory effect (Figure 1C).

**Figure 2:**
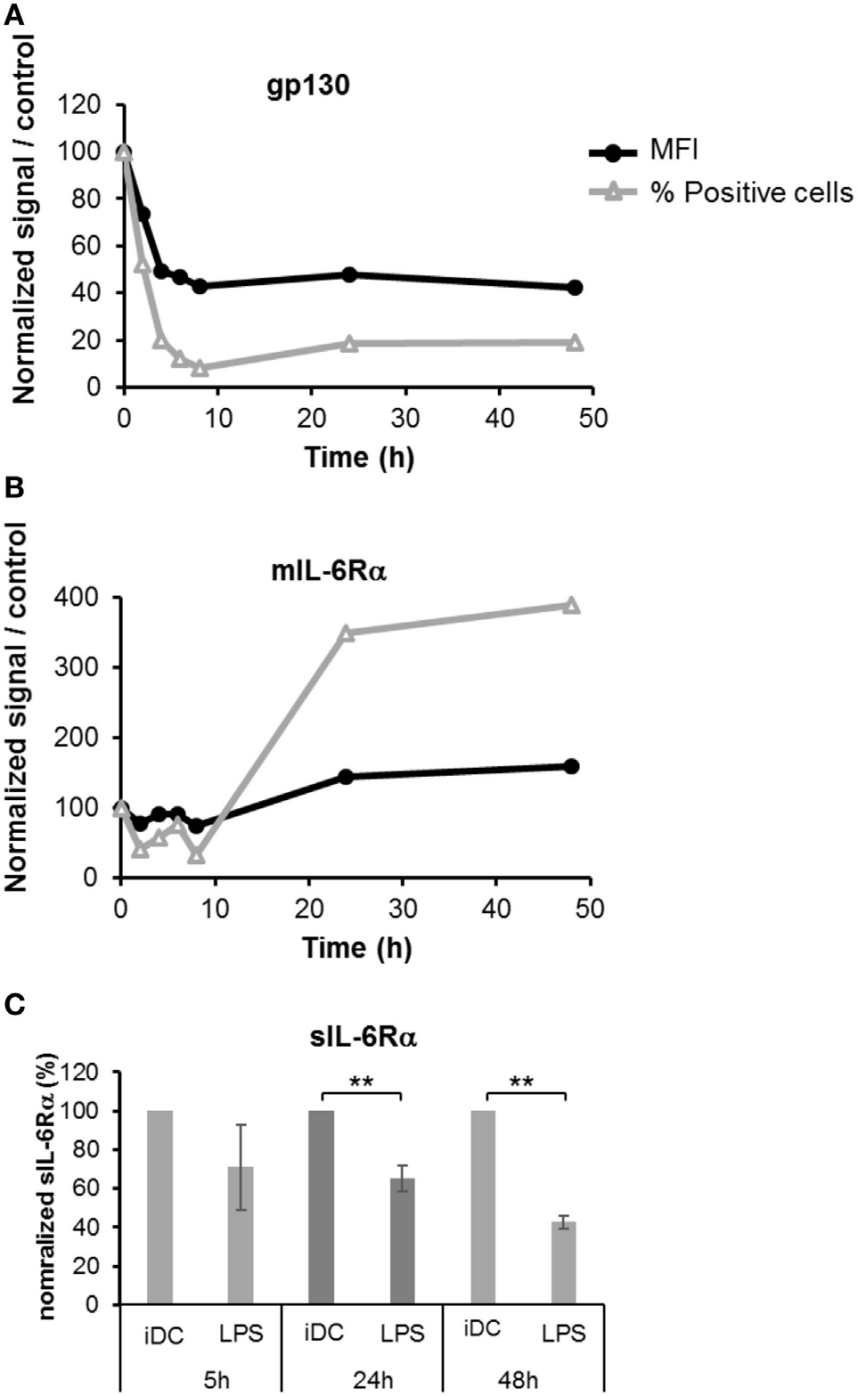
Kinetics of IL-6R subunits expression during the dendritic cells (DCs) maturation. iDCs were treated with 50 ng/ml LPS prior to staining with anti-gp130 **(A)** and anti-IL-6Rα antibodies **(B)**. The membrane expression level of each subunit was assessed by flow cytometry at 2, 4, 6, 8, 24, and 48 h. Results illustrate the mean fluorescence intensity (MFI, % of isotype staining) and the number of positive cells relative to control non-treated cells. **(C)** The supernatants of iDCs treated in panels **(A,B)** were analyzed by ELISA to measure the secretion of the soluble form of IL-6Rα. Results are relative to control non-treated cells ± SD. “**” indicates statistical difference with *p* < 0.01.

Different lengths of exposure of DCs to LPS (50 ng/ml) were tested and the levels of membrane bound gp130 and mIL-6Rα were assessed by flow cytometry. An important and rapid loss of gp130 expression was detected upon LPS maturation, and this loss was sustained over time (Figure 2A). Similarly, we observed a small but significant decrease of mIL-6Rα in the first hours, but this effect was fully reversed at 24 h and LPS-treated DCs sustainably over-expressed this subunit up to 48 h (Figures 1B and 2B). To evaluate the consequences of the increased mIL-6Rα expression following LPS maturation, we measured the concentration of sIL-6Rα in supernatants using an ELISA assay. The sIL-6Rα levels significantly decreased in LPS-matured DCs supernatants over time, showing that a higher membrane expression of IL-6Rα did not result in a higher secretion of sIL-6Rα (Figure 2C).

### Tocilizumab Induces Overexpression of both mIL-6Rα and sIL-6Rα but Not gp130

**Figure 3:**
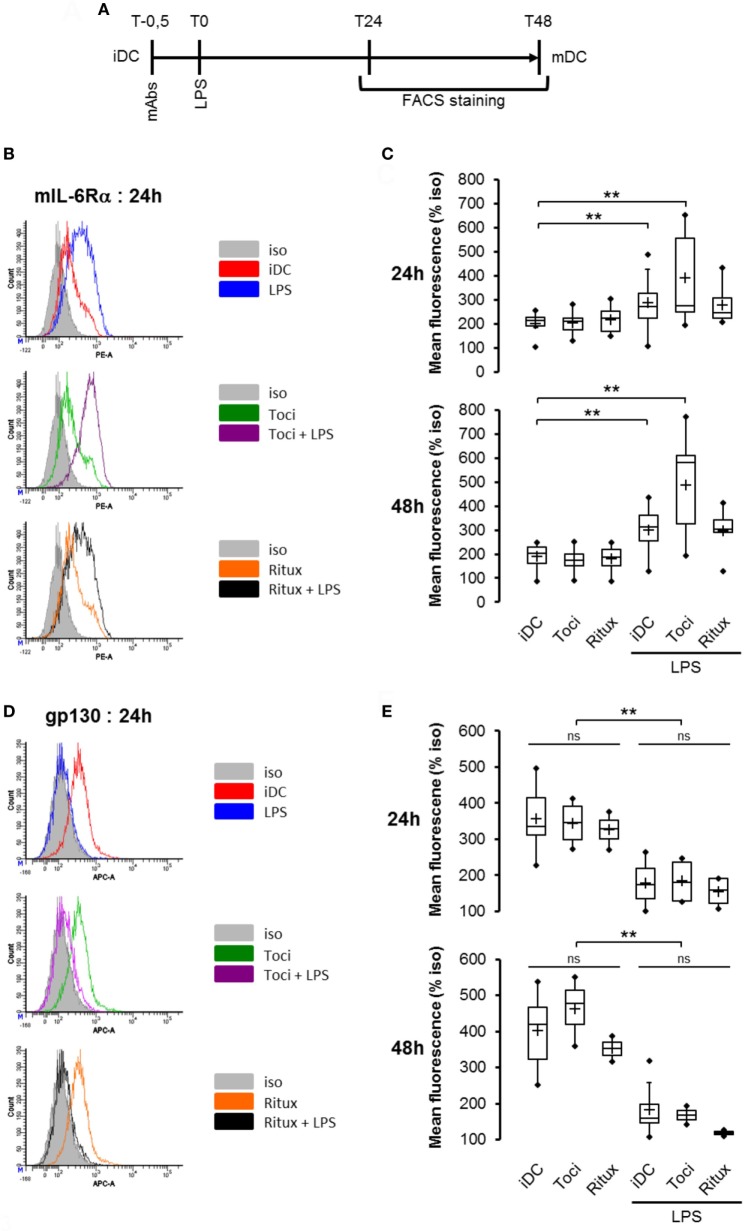
Tocilizumab induced overexpression of mIL-6Rα but not gp130. **(A)** Flowchart. iDCs were pre-treated for 30 min with tocilizumab or ritux 50 µg/ml prior to LPS maturation (50 ng/ml). Matured DCs were harvested at 24 and 48 h and analyzed by cytometry. mAbs stands for monoclonal antibodies. iDCs were treated as described in panel **(A)** and mIL-6Rα expression was assessed by cytometry **(B)** and results in different conditions are plotted in panel **(C)**. *n* ≥ 6. iDCs were treated as described in panel **(A)** and membrane gp130 expression was assessed by cytometry **(D)** and results in different conditions are plotted in **(E)**. *n* ≥ 3. “**” indicates statistical difference with *p* < 0.01.

In this model of LPS-induced DCs maturation, we investigated the effects of tociluzimab on IL-6R subunits. iDCs pretreated for 30 min with tocilizumab (toci) and rituximab (ritux) were subsequently exposed (or not) to LPS (Figure 3A). The anti-CD20 rituximab (ritux) is an IgG1 used here as a control antibody. As previously described in Figure 2, LPS induced a significant mIL-6Rα accumulation (Figure 3B,C) and membrane gp130 expression reduction (Figure 3D,E). Tocilizumab combined with LPS induced a higher mIL-6Rα expression compared to LPS alone (Figure 3B,C). Conversely, tocilizumab did not impact gp130 expression (Figure 3D,E).

**Figure 4:**
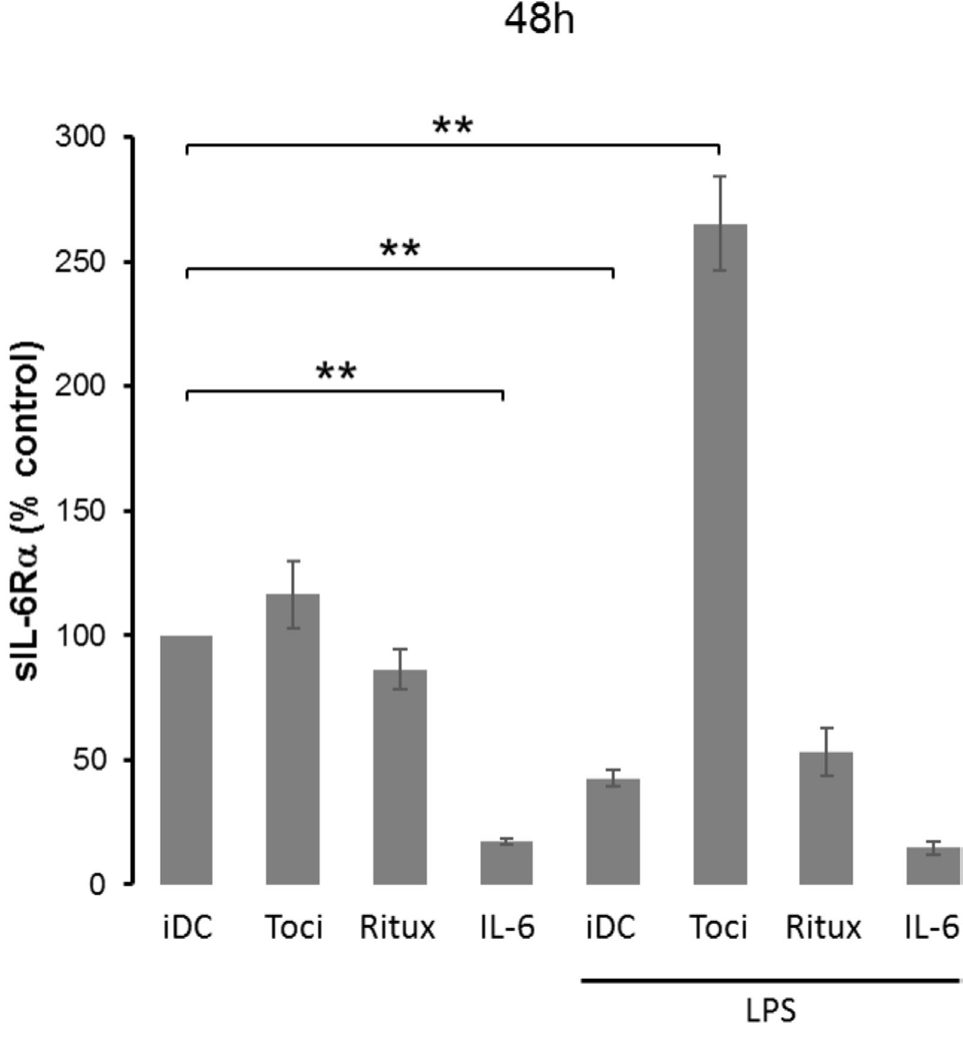
Tocilizumab increased IL-6 receptor α (IL-6Rα) secretion in lipopolysaccharide (LPS)-treated dendritic cells (DCs). iDCs were pretreated with IL-6 at 150 ng/ml or tocilizumab/ritux at 50 µg/ml for 30 min prior to LPS maturation (50 ng/ml). Supernatants were collected at 48 h and soluble form of IL-6Rα (sIL-6Rα) release was measured using an ELISA assay. Results are expressed as mean ± SD (*n* ≥ 4). “**” indicates statistical difference with *p* < 0.01.

Having showed that mIL-6Rα expression was modulated by therapeutic antibodies and LPS, we next questioned whether this was also the case for the soluble form sIL-6Rα. Following a similar protocol, supernatants were harvested and sIL-6Rα secretion was measured using an ELISA assay. As previously observed with mIL-6Rα, IL-6 alone, or in combination with LPS treatment induced a significant and sustained decrease in sIL-6Rα secretion in the supernatant (Figure 4). Interestingly, co-treatment of cells with tocilizumab and LPS not only increased mIL-6Rα expression (Figure 3B,C) but also sIL-6Rα secretion (Figure 4).

### Tocilizumab Does Not Inhibit IL-6 Secretion in LPS-Activated DCs

**Figure 5:**
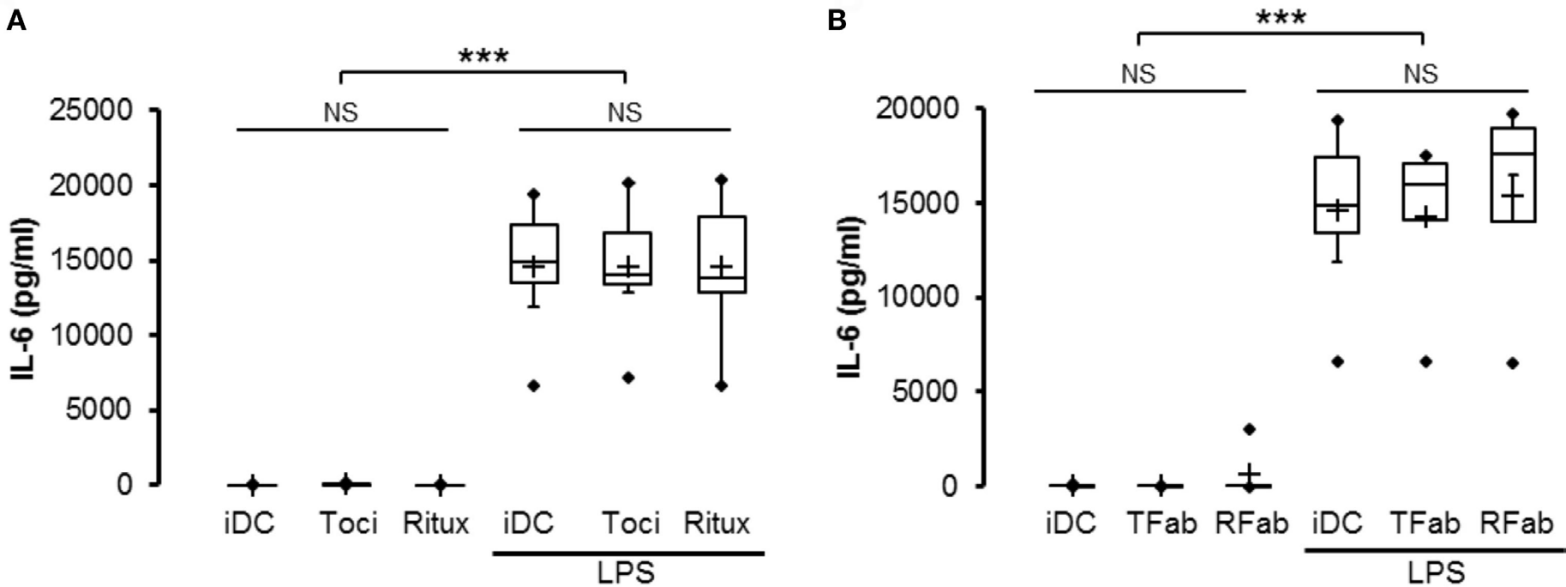
Tocilizumab did not inhibit IL-6 secretion of lipopolysaccharide (LPS)-activated dendritic cells (DCs). iDC were pretreated with the whole tocilizumab (toci) or rituximab (ritux) antibodies **(A)** or F(ab′)^2^ **(B)** of tocilizumab (TFab) or rituximab (RFab) (50 µg/ml) for 30 min prior to maturation with 50 ng/ml LPS during 48 h. ELISA assessed IL-6 secretion in supernatants. Results are expressed as mean ± SD (*n* = 8). “***” indicates statistical difference with *p* < 0.001.

As previously described, we showed that moDCs were able to secrete increased levels of IL-6 when maturated with 50 ng/ml LPS (Figure 5A). Treatment of human DCs with tocilizumab had no effect neither the IL-6 secretion nor IL-12, IFN-γ and TNFα (Figure S2 in Supplementary Material) in LPS-matured human DCs. Interestingly, when the cells were treated with the F(ab′)^2^ form of the tocilizumab (TFab), the results were equivalent to the whole antibody treatment regarding IL-6 secretion (Figure 5B).

### Tocilizumab Does Not Inhibit STAT3 Pathway in LPS-Matured DCs

**Figure 6:**
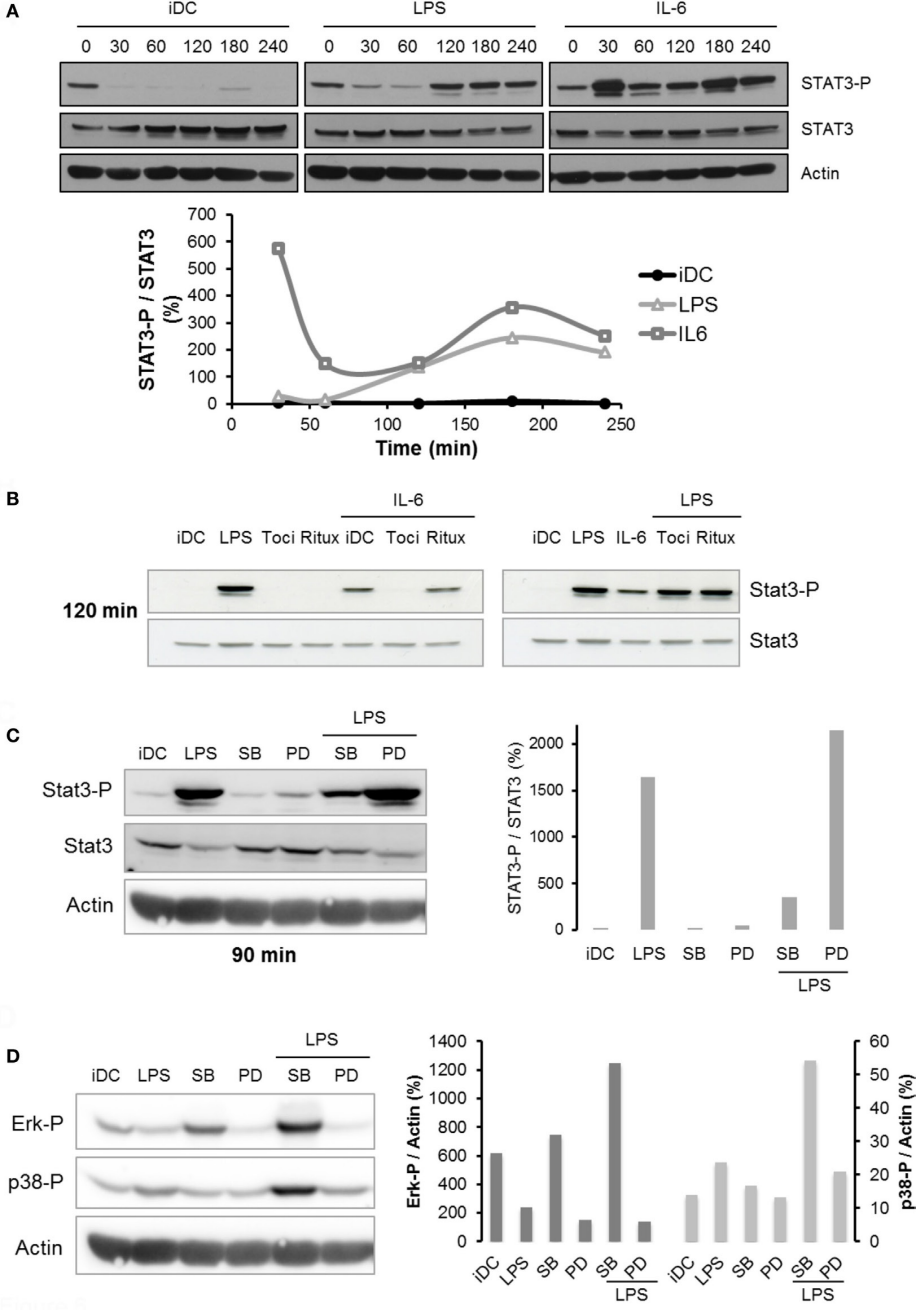
Regulation of STAT3 pathway following lipopolysaccharide (LPS) treatment. **(A)** iDCs were treated with LPS 50 ng/ml or IL-6 150 ng/ml. STAT3 and STAT3-Tyr705 phosphorylation levels were assessed by Western blot up to 240 min. The blot presented is representative of two independent experiments. Quantification of the bands was plotted as phosphorylated STAT3/non-phosphorylated STAT3 ratio. **(B)** iDCs were pretreated with tocilizumab or ritux 50 µg/ml during 30 min and then treated with LPS 50 ng/ml or IL-6 150 ng/ml. STAT3 and STAT3-Tyr705 phosphorylation levels were assessed by Western blot at 120 min. The blot presented is representative of two independent experiments. **(C,D)** iDCs were pretreated with SB203580 (SB) or PD98059 (PD) 25 µM prior to LPS maturation (50 ng/ml). STAT3 and STAT3-Tyr705 phosphorylation levels **(C)** and Erk1/2-Thr202/Tyr204 and p38-Thr180/Tyr182 phosphorylation levels **(D)** were assessed by Western blot. The quantification was done on the correct respective actin blots as explained in Section “Materials and Methods.” The blot presented is representative of two independent experiments. Quantification of the bands was plotted as phosphorylated STAT3/total STAT3 **(C)** or phosphorylated Erk1/2 or p38/actin **(D)** ratio.

Consistent with previously published articles (23, 24), IL-6 treatment resulted in a quick activation of the STAT3 pathway (Figure 6A) in a typical wave pattern as already observed with NF-κB or p53 pathway (25). Interestingly, LPS treatment also induced STAT3 phosphorylation in DCs, but this effect was significantly delayed compared to IL-6 treatment (120 min versus 30 min for IL-6) (Figure 6A). Thus, we questioned whether the STAT3 phosphorylation observed after LPS treatment could be the result of the LPS-induced IL-6 secretion. Figure 6B shows that IL-6-dependent STAT3 phosphorylation was completely reversed by addition of tocilizumab in the medium. Conversely, the LPS-activated STAT3 phosphorylation was not modified by tocilizumab (Figure 6B). This result suggests that LPS activates the STAT3 pathway independently of IL-6 secretion. We investigated whether MAPK pathway could be involved into the LPS-dependent STAT3 regulation in DCs and focused on MEK/Erk and p38 pathways using pharmacological inhibition with PD98059 and SB203580, respectively. Our results described an involvement of both pathways, but with opposite effects. Inhibition of MEK/Erk pathway with PD98059 increased STAT3 phosphorylation and potentiated LPS-induced activation. On the opposite, SB203580-induced p38 inhibition decreased the activation of STAT3 (Figure 6C; Figure S3 in Supplementary Material). Interestingly, LPS treatment induced an activation of p38 and an inhibition of Erk1/2 (Figure 6D). Surprisingly, inhibition of p38 MAPK also increased Erk phosphorylation in both iDCs and LPS-maturated DCs (Figure 6D).

### The MAPK Pathways Regulate mIL-6Rα Expression but Not gp130 Expression

**Figure 7:**
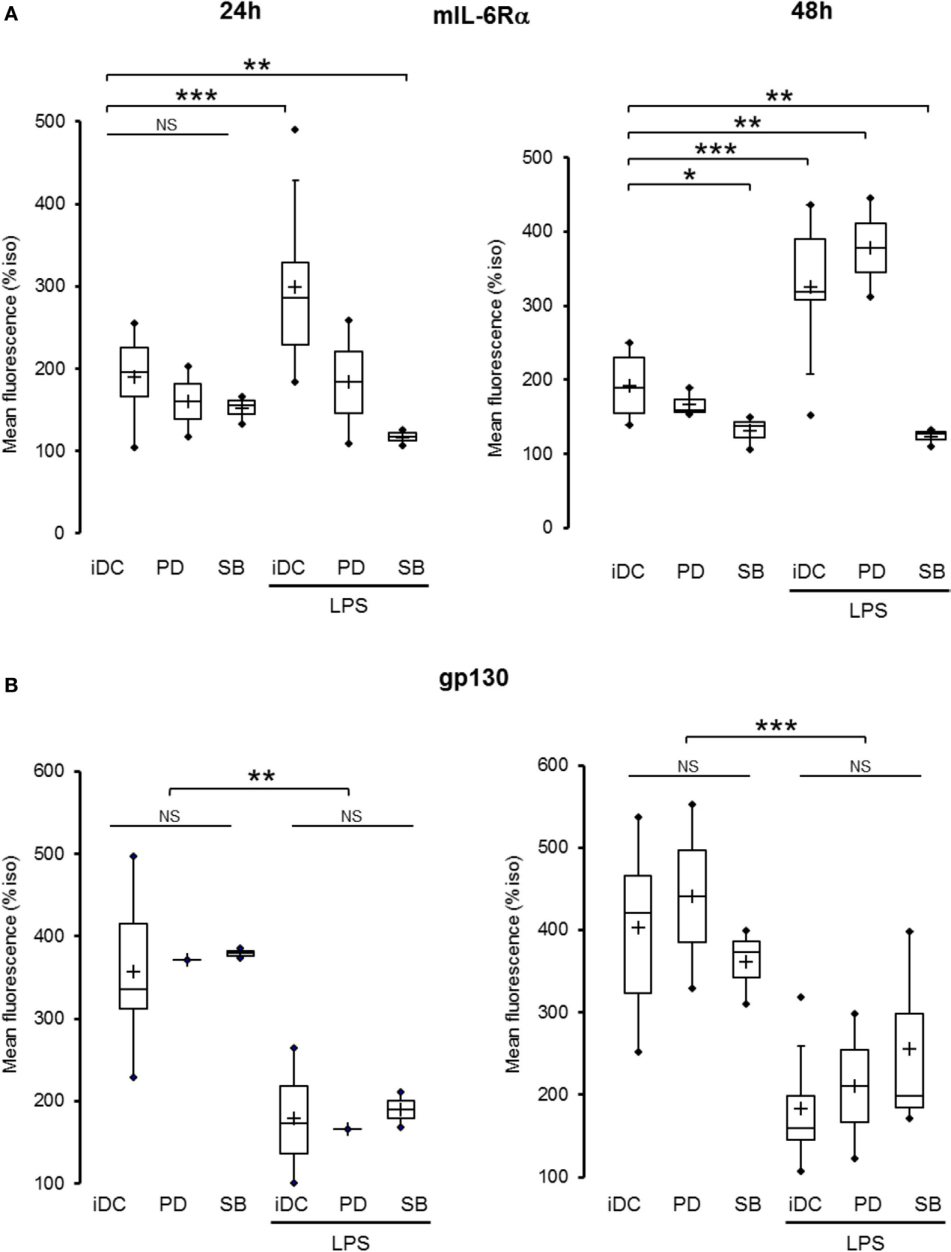
MAPK pathway regulated mIL-6Rα but not gp130 expression. iDCs were pretreated for 30 min with SB203580 (SB) or PD98059 (PD) 25 µM prior to lipopolysaccharide (LPS) maturation (50 ng/ml). Matured dendritic cells (DCs) were harvested at 24 and 48 h and analyzed by cytometry. **(A)** iDCs were treated as described above and mIL-6Rα expression was assessed (*n* ≥ 3). **(B)** iDCs were treated as described above and membrane gp130 expression was assessed (*n* ≥ 3). “*,” “**,” and “***” indicate statistical differences with, respectively, *p* < 0.05, *p* < 0.01, and *p* < 0.001.

Using flow cytometry, we assessed the effect of MAPK pathways activation on both IL-6R subunits expression. DCs co-treated with SB203580 and LPS displayed a significant reduction in mIL-6Rα expression compared to control at both 24 and 48 h (Figure 7A). Conversely, treatment with PD98059 potentiated the increase in LPS-induced mIL-6Rα expression at 48 h (Figure 7A). None of these inhibitors significantly modified gp130 expression (Figure 7B).

### LPS Maturation Induces DCs Resistance to IL-6-Dependent STAT3 Phosphorylation

**Figure 8:**
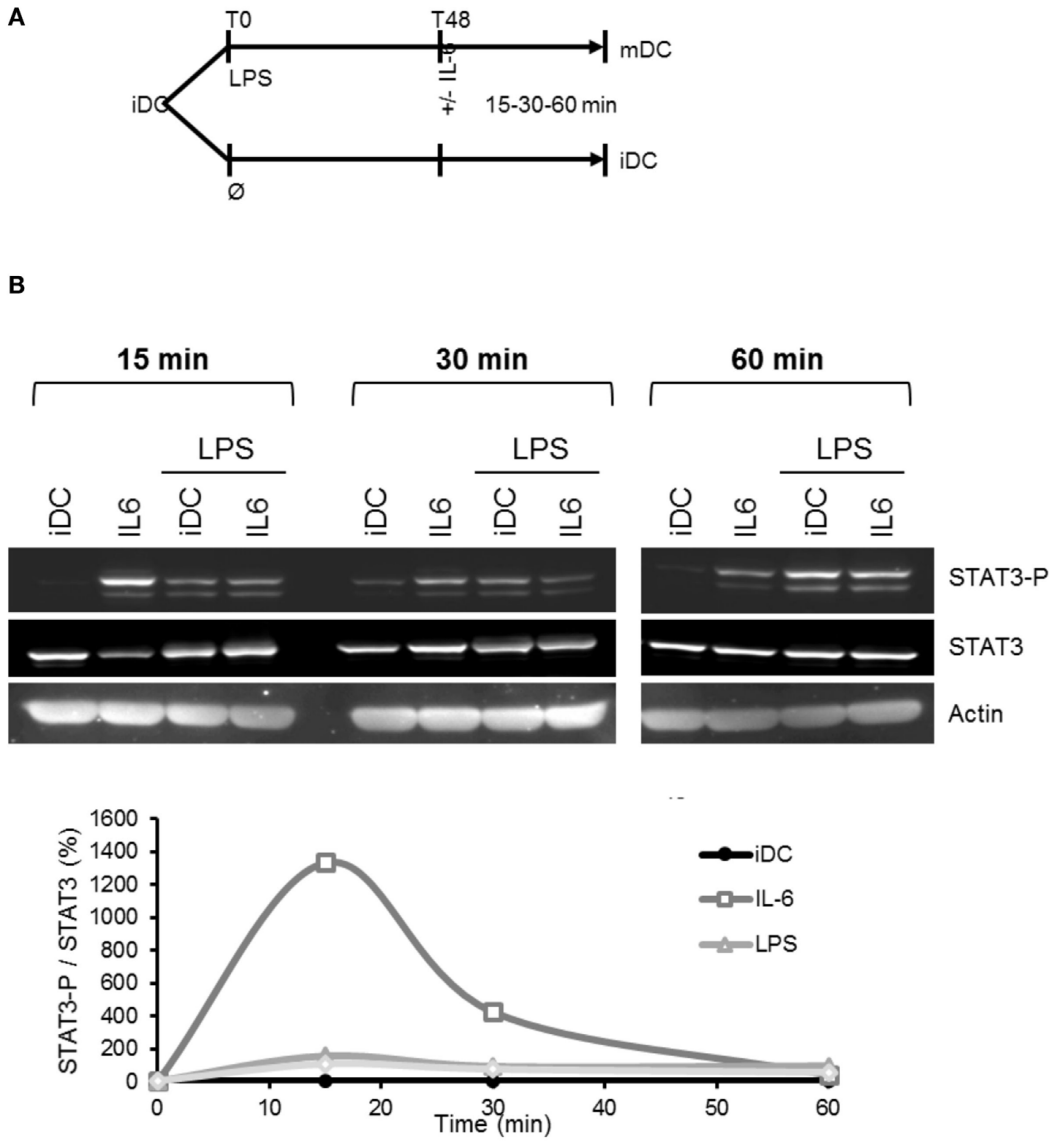
Lipopolysaccharide (LPS)-induced mIL-6R loss desensitizes dendritic cells (DCs) to IL-6 treatment. **(A)** Flowchart: iDCs underwent LPS maturation 50 ng/ml for 48 h prior to stimulation by IL-6 150 ng/ml. Analyses were made at 15, 30, and 60 min. Control cells were maintained in immature state before to be stimulated as well with IL-6. **(B)** iDCs were treated as described in **(A)**. STAT3 and STAT3-Tyr705 phosphorylation levels were assessed by Western blot. The blot presented is representative of three independent experiments. Quantification of the bands was plotted as phosphorylated STAT3/non-phosphorylated STAT3 ratio.

IL-6 secreted by DCs targets various cells such as T lymphocytes, thus modulating the Th17/Treg balance (26). However, the impact of this IL-6 secretion on DCs themselves was never determined. In our model, LPS-induced maturation of DCs resulted in a significant decrease in gp130 expression and an increase in mIL-6Rα expression (Figures 2A and 4). We, thus, assessed whether these DCs could still respond to IL-6 stimulation. Addition of IL-6 on LPS-matured DCs did not result in any significant change in STAT3 phosphorylation compared to control immature cells (Figure 8).

## Discussion

Considering the large use of tocilizumab in the treatment of several inflammatory diseases, the question of its effect on DCs should be raised, given their major importance in inflammation and inflammatory diseases.

Like almost all cytokines, IL-6 induces a negative feedback on its receptor expression (13, 27). The regulation of both IL-6R subunits remains debated in many different cellular models, including monocytes, but no one ever reported about this regulation in DCs. We showed that addition of IL-6 on DCs strongly and sustainably decreased membrane and soluble IL-6Rα as well as gp130 subunit expressions. DCs are, therefore, able to respond to IL-6 stimulation and to desensitize themselves through the downregulation of both IL-6R subunits expression.

In juvenile idiopathic arthritis, gp130 expression was reduced in monocytes and dependent on p38 MAPK activation (28) while it was increased in CD4^+^ T cells in synovial tissue (but not in synovial fluid). This increase in T cells was dependent on IL-10 secretion (29). Thus, each cell in the joint would have a specific regulation of gp130 depending on its microenvironment. Regarding human LPS-DCs, we showed that gp130 expression was reduced following LPS maturation. Our data in human DCs echo with those of Wang et al. (30). The absence of effect of tocilizumab on the LPS-induced decrease in gp130 expression might indicate that its down regulation is independent of IL-6 signaling. Moreover, neither p38 MAPK inhibitor nor Erk inhibitor were able to modify gp130 expression in both iDCs and LPS-matured DCs. The regulation of the gp130 subunit expression appears to be more complex than usual cytokine modulation and seems to depend on tissue and inflammatory conditions without clear-cut pathways evidenced.

Another major point in our study is the regulation of IL-6Rα expression. We showed a time-dependant differential regulation following DC activation. First, we observed a significant down regulation of mIL-6Rα immediately following LPS treatment, which was sustained for at least 8 h. These results correlate with the observations done in hepatocytes (13). Second, we assessed from 24 h an increased expression of mIL-6Rα on the surface of LPS-activated DCs, which was potentiated by the presence of tocilizumab. Conversely, we measured an important and sustained decrease of sIL-6Rα release by LPS-treated DCs. Interestingly, tocilizumab treatment potentiates the mIL-6Rα expression as well as sIL-6Rα release. We could hypothesize that tocilizumab stabilizes the expression of mIL-6Rα to the membrane, increasing its membrane half-life, which finally results in an increased release of the sIL-6Rα subunit.

The complexity of IL-6 regulation comes from the fact that there is a membrane and a soluble form for both gp130 and IL-6Rα. The respective role of each form remains unclear (31, 32). As explained above sIL-6Rα is essential to the *trans*-signaling and soluble gp130 plays an antagonist role in IL-6 regulation by chelating the sIL-6Rα. Inflammatory DCs in the presence of tocilizumab expressed and secreted both forms of IL-6Rα without being able to respond to IL-6 stimulus (Figure 8).

Regarding soluble IL-6Rα secretion, Xu and Derynck showed in various cell lines that activation of ADAM17 was p38-dependent, resulting in an increase of the shedding activity (33). This mechanism does not probably occur in our model because the LPS-treated cells displayed a decrease of sIL-6Rα release despite an effective p38 MAPK activation (Figures 2C and 4). The mechanisms of this sIL-6Rα regulation in LPS-treated DCs deserve to be thoroughly explored. Altogether, tocilizumab makes DCs a large source of IL-6Rα secretion that might contribute to their inflammatory status.

In moDCs and myeloid cells, the STAT3 pathway is considered a negative regulatory pathway as numerous data support the fact that STAT3 activation inhibits DCs maturation (14). Our data showed that LPS activates STAT3 in a MAPK-dependent manner as shown by others (34). In our model, LPS induced a delayed STAT3 phosphorylation compared with IL-6. LPS likely activated STAT3 through an indirect mechanism (Figure 6A). Tocilizumab did not inhibit the LPS-induced STAT3 phosphorylation in DCs, as shown in Figure 6B. Serial signal transduction pathways triggered by TLR4 activation mediate the activation of MAPK and STAT3. Previous work suggested that the IL-6/gp130/STAT3 axis was responsible for the modulation of the LPS/TLR4 inflammation in a murine model (35) but the opposite situation of LPS modulating the IL-6 axis in DCs could be a better representation of the real situation in inflammatory diseases.

In our model, p38 MAPK and Erk inhibitors significantly modulated STAT3 phosphorylation in an opposite way. This suggests that MEK/Erk has an inhibitory effect on STAT3 activation and its inhibition with PD98059 could reinforce the LPS-dependent effect. Conversely, p38 positively controls STAT3 activation and its inhibition with SB203580 abolishes the p38-dependent STAT3 induction. In addition, we showed that SB203580 could activate Erk phosphorylation (Figure 6D), an unspecific effect already described (36) that could potentiate the MEK/Erk-dependent inhibitory effect on STAT3 (see Figure S3 in Supplementary Material).

In this study, we showed that tocilizumab had no effect on LPS-induced IL-6 secretion in human matured DCs. This point is of utmost importance because it suggests that DCs could remain a significant source of IL-6, contributing to the inflammation and joint destruction in the presence of tocilizumab in RA patients. This large amount of IL-6 could activate all cells expressing gp130 in the joint. Moreover the large amounts of both membrane and soluble forms of IL-6Rα could also participate in the inflammation circle existing in RA. Inflammatory DCs have been found infiltrating the synovium, and supporting a high Th1 response (37, 38). Treatment with tocilizumab exacerbated the circulating mDCs reduction (39), without one knowing whether it contributes to mDCs infiltration in joints. Everything happens in the synovium where inflamed mDCs with low CCR7 expression could not escape and where they could secrete large amount of IL-6 and sIL-6Rα, enhancing the inflammatory circle of IL-6 on synovial cells. Thus, inflamed mDCs might be key actors in RA physiopathology. There is an important inter-individual variability of response to tocilizumab. Only 30% patients display a remission, 20% of which do not respond anymore after 24 weeks (40). Two hypotheses may explain these observations: (1) a variability in the relation between the dose administered and blood concentration (PK) and/or (2) a variability in the relation between blood concentration and therapeutic response (PK/PD). Without ignoring these possibilities, the mechanism of resistance to tocilizumab suggested by our results may be central in the absence of response or poor response to tocilizumab observed. Consequently, in order to improve the treatment efficiency, it appears particularly relevant to thoroughly analyze the conditions in which a patient could develop this IL-6Rα upregulation and whether this process is effectively linked to the development of tocilizumab resistance in RA patients. Physicians would then possess an interesting biomarker to adjust properly the treatment strategy.

Finally, our results raise two questions: would we have similar results using another anti-IL-6Rα such as sarilumab (currently in phase III clinical trials in RA treatment)? Would it be relevant to use an anti-IL-6 antibody (e.g., siltuximab) in RA patients displaying or developing a poor response to tocilizumab?

Upon LPS maturation, DCs secrete IL-6, upregulate mIL-6Rα expression, but downregulate gp130 expression, with consequently a loss of sensitivity to IL-6 signaling. The treatment of these inflammatory DCs with tocilizumab has an exacerbated effect on sIL-6Rα secretion. While there are many on-going clinical trials exploring the efficacy/safety of tocilizumab (41), we believe that systematic and deep analyses of the impact of this antibody on the main cells involved in RA, such osteoclasts, osteoblasts, and immune cells are necessary to better define the parameters of its clinical use.

## Author Contributions

DM co-elaborated the project, designed and performed the experiments, and analyzed the results. AH trained DM in flow cytometry experiments and performed some Western blot and cytometry experiments. FI was involved in discussion about analysis and interpretation of the results and also brought its medical expertise. FV-R co-elaborated the project, supervised the design and the analyses of experiments. DM, FI, VG-G, and FV-R wrote the article.

## Conflict of Interest Statement

The authors declare that the research was conducted in the absence of any commercial or financial relationships that could be construed as a potential conflict of interest.
