# Supplementary figures and images for: Tocilizumab Contributes to the Inflammatory Status of Mature Dendritic Cells through Interleukin-6 Receptor Subunits Modulation
Source: Front Immunol. 2017 Aug 16;8:926. doi: 10.3389/fimmu.2017.00926 (PMC5561017; doi:10.3389/fimmu.2017.00926)

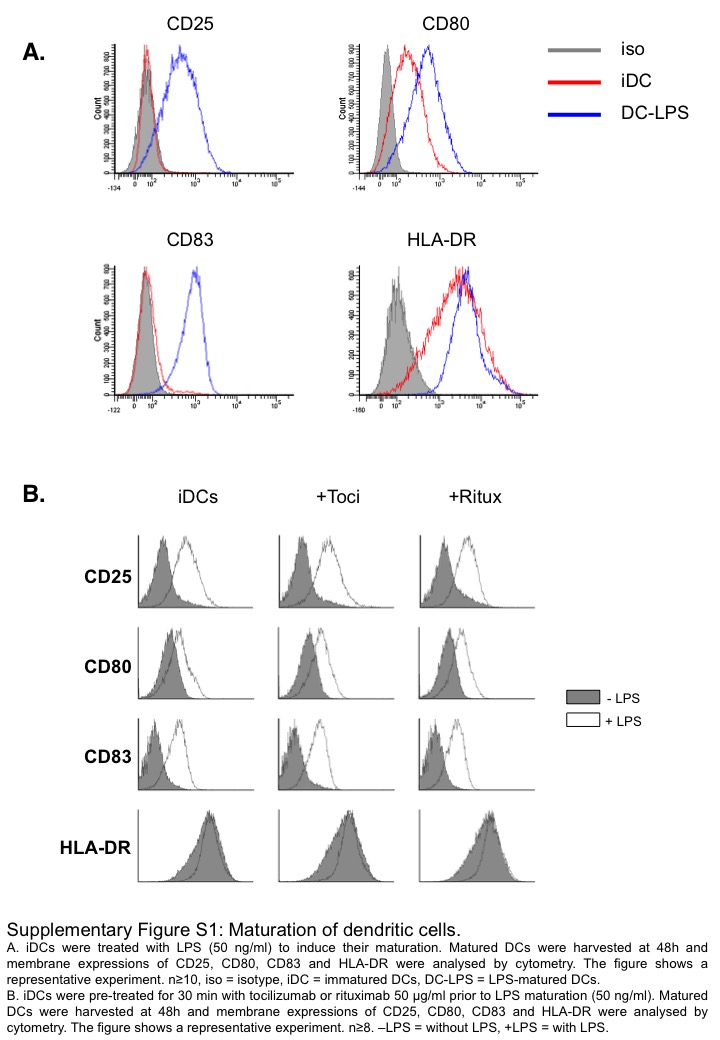

Supplement: Supplementary file 1 [file image_1.jpeg]

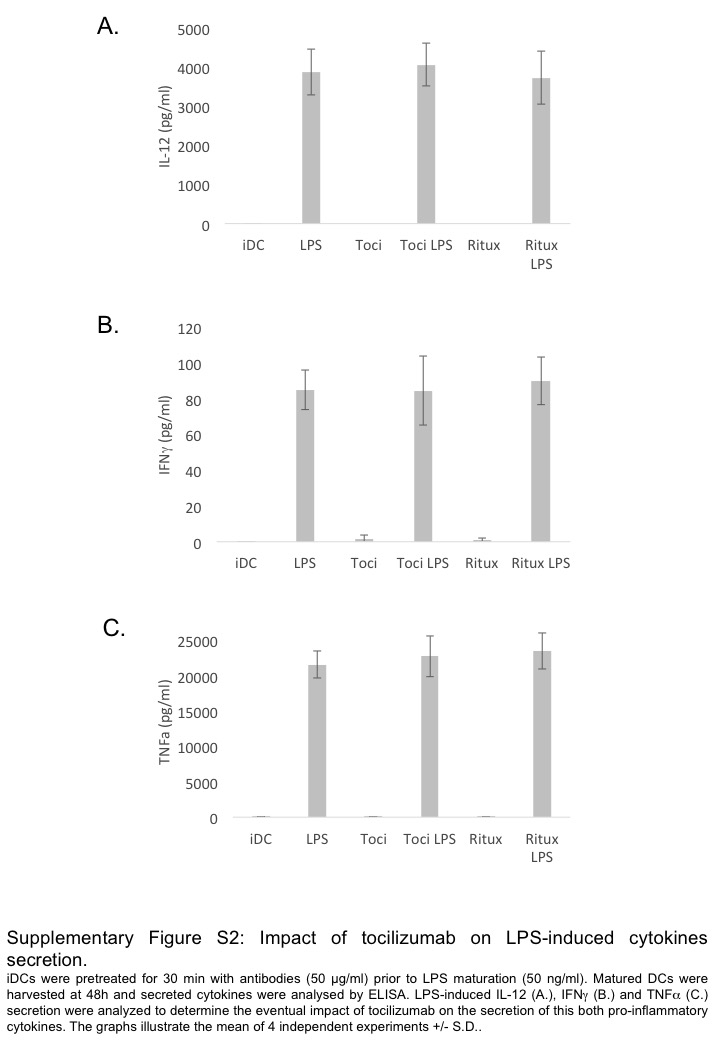

Supplement: Supplementary file 2 [file image_2.jpeg]

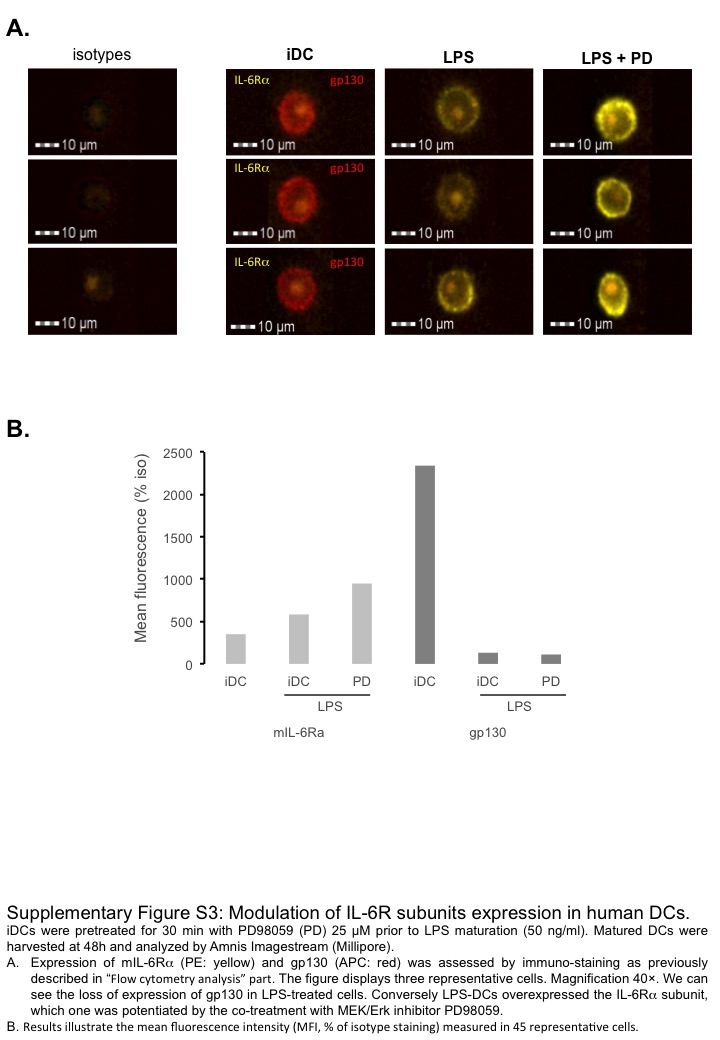

Supplement: Supplementary file 3 [file image_3.jpeg]
